# Genome-wide association study identifies candidate genes for piglet splay leg syndrome in different populations

**DOI:** 10.1186/s12863-017-0532-4

**Published:** 2017-07-05

**Authors:** Xingjie Hao, Graham Plastow, Chunyan Zhang, Sutong Xu, Zhiqiu Hu, Tianfu Yang, Kai Wang, Huawei Yang, Xiaoxue Yin, Shili Liu, Zhenghua Wang, Zhiquan Wang, Shujun Zhang

**Affiliations:** 1 Key Lab of Breeding and Reproduction of Ministry of Education, Huazhong Agricultural University, Wuhan, Hubei 430070 China; 2 Livestock Gentec Center, Department of Agricultural, Food and Nutritional Science, University of Alberta, Edmonton, AB T6G 2C8 Canada; 3 Hubei Tianzhong Stock Corporation, Wuhan, Hubei China

**Keywords:** Genome-wide association study (GWAS), Pig, Splay leg, Principal component analysis (PCA), Muscle

## Abstract

**Background:**

Piglet splay leg syndrome (PSL) is one of the most frequent genetic defects, and can cause considerable economic loss in pig production. The present understanding of etiology and pathogenesis of PSL is poor. The current study focused on identifying loci associated with PSL through a genome-wide association study (GWAS) performed with the Illumina Porcine60 SNP Beadchip v2.0. The study was a case/control design with four pig populations (Duroc, Landrace, Yorkshire and one crossbred of Landrace × Yorkshire).

**Result:**

After quality control of the genotyping data, 185 animals (73 cases, 112 controls) and 43,495 SNPs were retained for further analysis. Principal components (PCs) identified from the genomic kinship matrix were included in the statistical model for correcting the effect of population structure. Seven chromosome-wide significant SNPs were identified on *Sus scrofa* chromosome 1 (SSC1), SSC2 (2 SNPs), SSC7, SSC15 (2 SNPs) and SSC16 after strict Bonferroni correction. Four genes (*HOMER1* and *JMY* on SSC2, *ITGA1* on SSC16, and *RAB32* on SSC1) related to muscle development, glycogen metabolism and mitochondrial dynamics were identified as potential candidate genes for PSL.

**Conclusions:**

We identified seven chromosome-wide significant SNPs associated with PSL and four potential candidate genes for PSL. To our knowledge, this is the first pilot study aiming to identify the loci associated with PSL using GWAS. Further investigations and validations for those findings are encouraged.

## Background

Piglet splay leg (PSL) syndrome is one of the most frequent genetic defects in commercial pig production [1]. It is characterized by impaired ability to stand and walk after birth [2]. The hind legs of PSL are splayed sideways and in severe cases the forelegs were also affected [3]. PSL is regarded as a considerable source of economic losses in pig production, due to the fact that the affected piglets unable to walk freely are often crushed by the sow or die from starvation, which could account for about 50% of the total loss among affected piglets [4]. Generally, lean type breeds are frequently affected and the incidence in male piglets is higher than that in females [5–7]. The distribution of muscle fibers in PSL was found to be different from normal animals. A specific distribution pattern of smaller fibre size and higher fibre density in the semitendinosus, longissimus dorsi and gastrocnemius muscles was identified [4], and longissimus dorsi was also found to be the most frequently and evidently affected muscle [8].

Etiology and pathogenesis of PSL is complex, and predisposing factors were thought to include genetic and environmental factors, such as nutrition, management, pharmacological administration, and mycotoxins [4]. Porcine reproductive and respiratory syndrome virus (PRRSV) could increase the number of stillborn, weak, and light weight as well as splay-legged piglets [9]. Histomorphological investigations described PSL as myofibrillar hypoplasia, however, which was not exclusive to PSL as this condition was also found in clinically normal piglets [10]. In addition, ultrastructural analysis of PSL muscles clearly demonstrated an increased accumulation of glycogen compared with the muscles of normal piglets [11]. Besides the histological studies, the expression of the atrophy marker *FBXO32* highly increased in PSL muscles (semitendinosus, longissimus dorsi, and gastrocnemius muscles) [4]. However, this expression result for *MAFbx* (alias for *FBXO32*) was not fully confirmed in later research [12]. Maak et al. [13] compared genome wide gene expression of the hind leg muscles (M.adductores, M.gracilis, M.sartorius) from affected piglets and their healthy littermates using GeneChip® Porcine Genome Array. However no significant differences were found using a standard paired t-test, only four genes (*SQSTM1, SSRP1, DDIT4, MAF*) with significant (Wilcoxon, *p* < 0.05) differential expression levels in at least two muscles were found after using a sum rank test and suggested further investigation. In their following study [14, 15], another two differentially expressed gene *ITGA5*, *ZDHHC9* were also excluded as candidate genes for PSL.

Despite the fact that PSL is a source of considerable economic loss in pig production, up till now, the genetic mutations related to PSL are still unknown. The microarray studies provide some potential targets, however, they only measure the relative abundance of predefined transcripts. In addition, the timing of samples may impact the results especially as the muscle transcriptome is very dynamic during piglet development [16, 17]. It is also unclear what period of development is related to a PSL outcome. Therefore, GWAS may be a better strategy than the comparative transcriptome analysis to identify the causative genes and mutations for PSL. GWAS using a case/control design has successfully identified multiple loci for complex diseases and traits in livestock [18–23] during the last decade. Motivated by further clarifying the genetic basis of PSL, we conducted a pilot investigation by GWAS to identify the potential genomic regions and genes affecting PSL using a case/control design.

## Methods

### Animals and data collection

Animals used in this study were raised at the Hubei Tianzhong Stock Corporation (Hubei, China) from January 2012 to October 2013. The farm is one of the national core pig breeding farms and part of the China swine genetic improvement program. According to the farm’s production records, PSL piglets were observed in more than 1% of newborn piglets, especially in male piglets during that period. The farrowing sows and piglets were kept in farrowing crates before weaning. The PSL affected animals were defined as having the hind legs of the piglets splayed sideways showing an impaired ability to stand and walk 24 h after birth. The ear tissues of 192 pigs, including 76 affected and 116 normal were collected from four populations (Duroc, Landrace, Yorkshire and one crossbred of Landrace × Yorkshire) by using ear punches. When one affected piglet was collected, one to three the same breed normal piglets with similar weight and size by visual assessment from the affected or the other normal litters were also collected at the same time. The 76 affected piglets were collected in 53 affected litters and the number of sampled cases in each affected litter ranged from 1 to 5. For the 116 normal animals with similar birthdate to the affected piglets, 47 animals were from 47 affected litters and 69 animals were from 69 normal litters which had common ancestors with the affected litters tracking back 2 ~ 5 generations.

### Genotyping and quality control

Ear tissues for 192 animals were collected and stored in 75% alcohol for DNA extraction. DNA was extracted using a phenol/chloroform method and diluted to a final concentration of 50 ng/μL. Genotyping was conducted at Delta Genomics (Edmonton, AB, Canada) using Illumina PorcineSNP60 v2 Genotyping BeadChip. Only the SNPs mapped in the Sscrofa 10.2 genome assembly were used.

The quality control of genotype of 192 animals was performed using PLINK v1.07 [24] to remove SNPs with a call rate less than 90%, a minor allele frequency (MAF) less than 0.01 and a significant deviation from Hardy-Weinberg equilibrium (*P* ≤ 10^−5^). Moreover, animals with more than 10% missing genotypes were removed from the data set. After quality control, a final set of 43,495 SNPs from 185 animals (73 cases vs. 112 controls) were retained for further statistical analysis (see Table 1).

**Table 1:** Sample information for GWAS

	Normal-Male	Normal-Female	Affected-Male	Affected-Female	Total
Yorkshire	18	49	34	3	104
Duroc	8	20	10	11	49
Landrace	4	8	5	2	19
Crossbreed	0	5	6	2	13
Total	30	82	55	18	185

### GWAS analysis

After quality control, pairwise kinship was estimated using genome-wide autosomal SNP information by *ibs* function in the GenABEL package [25]. Then principal components (PCs) derived from genomic kinship matrix were used to correct population structure arising from different populations used in the study. Based on Kaiser’s Criterion [26], the first six PCs with eigenvalues bigger than one (14.96, 4.73, 1.68, 1.38, 1.23, 1.07) were selected as fixed factors for correcting the population structure. By logistic regression, sex was found to be significant (*P* < 10^−9^) for the development of PSL and selected as another fixed factor. Eventually the GWAS was performed by applying principal component analysis (PCA), which is known as EIGENSTRAT [27] and implemented in the GenABEL package [25] by using *egscore* function. The statistical model was: Y = S+(PC_1_ + PC_2_+ … + PC_6_) + SNP_i_ + e, where Y is a vector of phenotypic values, S is a vector of sex, PC_i_ is ith PC vector, SNP_i_ is the genotype vector for SNP_i_, and e is a vector of residuals.

In consideration of multiple testing, the significance level was corrected using the Bonferroni method. The genome-wide significance threshold was set at *P* < 0.05/N (0.05/43,495 = 1.15 × 10^−6^), where N is the total number of SNPs tested in the analysis after quality control. While the chromosome-wide significance threshold was set at *P* < 0.05/M (0.05/5007 = 9.98 × 10^−6^), where M is the max number of SNPs among each chromosomes (SSC 1 had the max number of SNPs (5007)) after quality control.

The bioinformatics databases Ensembl (http://www.ensembl.org/) and KEGG (http://www.genome.jp/kegg/) were used for the candidate gene screening. Only the nearest genes to the significant SNPs were considered in view of linkage disequilibrium (LD) decay as the distance between marker and QTL increased [28–32].

## Results and discussion

### Phenotype and SNP characteristics after quality control

In this study, the affected animals were characterized by the hind legs splaying sideways and the forelegs being normal, in accordance to the fact that some similar traits of the hind leg and fore leg structure could be classed into different categories [33]. After quality control of the genotypes, a final set of 185 animals (73 cases vs. 112 controls) remained for further statistical analysis (see Table 1). The remaining 185 animals were from four populations: Yorkshire, Duroc, Landrace and a Crossbred of Yorkshire and Landrace. Among 73 cases, there were more male piglets than male (55 males vs. 18 females), which was consistent with the fact that the farm’s production records indicating male piglets with higher risk of being affected and the previous investigations [5–7]. Logistic regression analysis proved that sex was a significant factor (*P* < 10^−9^). Moreover, androgenic steroids including testosterone could increase skeletal muscle mass and growth [34, 35], thus indicating that sex was an important factor contributing to the development of PSL.

After quality control, a final set of 43,495 SNPs including 42,462 autosomal SNPs were retained, thus providing a uniform genome-wide coverage with mean spacing of 59.5 kb and median spacing of 33.3 kb.

### Population structure

**Fig. 1:**
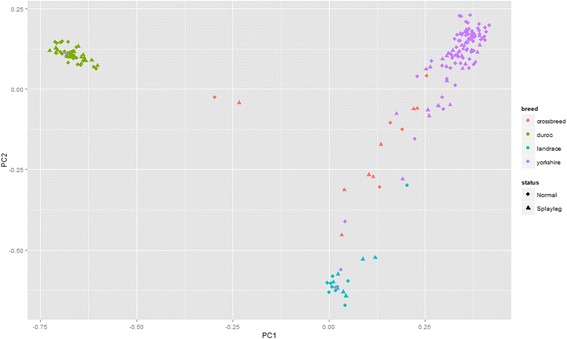
The top two axes of variation of experiment animal samples

Pairwise kinship was estimated using the genome-wide 42,462 autosomal SNPs. The first two PCs derived from genomic kinship matrix were shown in Fig. 1. Clusters were detected and corresponded to the different populations. It was noted that one Yorkshire individual was clustered with the Duroc population and another two Yorkshire individuals were clustered with the Landrace population, which might be breed registry errors in the farm. In addition, two crossbred pigs were not clustered with any populations, which might result from the use of the wrong breeds during intercross mating. However, no irregular distribution of the affected/unaffected animals was seen throughout the clusters. In this study, PCs were preferred to be used for population structure correction rather than directly using breeds to separate the animals into definitely discrete groups, since PCs could infer continuous genetic variations in the sample population which had some relationship between individuals [27]. Moreover, PCs were less influenced by the connections between the breeds (crossbreed of Landrace × Yorkshire) and several breed registry errors. Because of the incomplete pedigree information and different populations in the study, genomic kinship was used for PCA for the higher correctness and accuracy than pedigree. More importantly, the genomic kinship could connect and compare multiple breeds or lines, which could not be connected when the pedigree is unclear or unknown [36, 37].

For GWAS, one of the most important sources of false positive association was a sample consisting of a mixture of breeds [32]. Fortunately this problem could be avoided by fitting the statistical model with the known breeds [38] or the PCs [37]. both of which could correct the population structure, while we ignored this problem in our previous study [39]. It should be noted that the top PCs could reflect family relatedness [40], although PCA could not explicitly model family structure and cryptic relatedness compared with a mixed linear model. Given that mixed linear model association could suffer a severe loss of power due to case/control ascertainment [41], PCA was used in this GWAS instead of mixed linear model association.

### Potential genes identified by GWAS

**Fig. 2:**
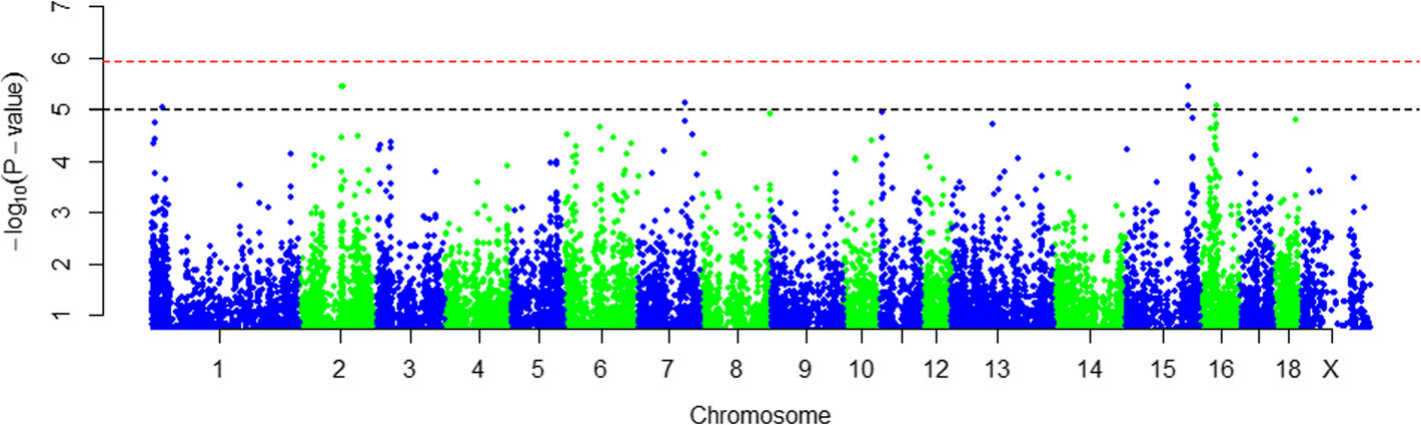
Manhattan plot of the genome-wide association study with PSL. SSC1–18 and SSCX are shown in different colors. The *red horizontal dash line* indicates the genome-wide significance level, and the *black dash line* indicates the chromosome-wide significance. Two chromosome-wide significant SNPs on SSC2 overlapped in the figure

The association analysis was performed for 43,495 SNPs distributed over SSC1–18 and SSCX. The genome-wide and chromosome-wide significant thresholds were set at 1.15 × 10^−6^ (=0.05/43,495) and 9.98 × 10^−6^ (=0.05/5007) respectively according to Bonferroni correction. The Manhattan plot of the –log10 based *p*-values was presented in Fig. 2. In total, seven chromosome-wide significant SNPs were identified on five autosomes (SSC1, SSC2, SSC7, SSC15 and SSC16), while no SNPs exceeded the genome-wide significant threshold. In this study, the Bonferroni method was used as a very strict correction for multiple testing; consequently, the chromosome-wide significant SNPs should also be taken into consideration. Chromosome-wide significant SNPs and the nearest genes were shown in Table 2.

**Table 2:** Chromosome-wide significant SNPs for PSL (new)

SNP	Location (SSC:bp)	MAF (case/control)	Nearest gene	Strand	Distance to the nearest genes (bp)	Raw *p* value
ASGA0001519	1:21,382,287	0.461/0.455	*RAB32*	−	40,041	9.32E-06
ALGA0014296	2:89,825,751	0.240/0.455	*JMY/ HOMER1*	+/−	15,695/18609	3.61E-06
ALGA0014308	2:89,995,349	0.240/0.455	*HOMER1*	−	0	3.61E-06
H3GA0022494	7:98,558,186	0.253/0.107	*ENSSSCP00000021921*	+	235,866	7.31E-06
ALGA0087037	15:131,897,923	0.356/0.482	*TNP1*	−	97,522	3.64E-06
MARC0003725	15:131,919,478	0.445/0.420	*TNP1*	−	119,107	8.44E-06
MARC0006026	16:33,866,754	0.323/0.205	*ITGA1*	+	74,317	8.71E-06

#### HOMER1

The two most significant SNPs ALGA0014296 (at 89.8 Mb) and ALGA0014308 (at 90.0 Mb) on SSC2 are located in a 170 kb high LD block (r^2^ = 1), which covered the *HOMER1* gene. According to the Pig EST Data Explorer (PEDE, http://pede.dna.affrc.go.jp/), the *HOMER1* gene expresses in pig longissimus [42]. The longissimus was reported to be the most frequently and evidently affected muscle of PSL by histochemical study [8]. The function of *HOMER1* in pigs has not been fully researched, however in Zebrafish embryos *HOMER-1b* was the target gene of *MicroRNA-3906* which regulated fast muscle differentiation and calcium homeostasis [43]. In addition, Stiber et al. [44] showed that mice lacking *HOMER1* exhibited a myopathy characterized by decreased fiber cross-sectional area and skeletal muscle force generation. Moreover, they also observed a significant decreased expression for *HOMER1* in the mouse model of Duchenne’s muscular dystrophy, which is a recessive X-linked form of muscular dystrophy resulting in muscle degeneration and premature death [45]. Furthermore, KEGG pathway analysis indicated that *HOMER* is involved in the *FOXO* signaling pathway, which regulated muscle atrophy, glycolysis/gluconeogenesis metabolism, and apoptosis. On the basis that PSL was characterized by decreased muscle fiber size in muscles [4], then the *HOMER1* gene could be regarded as a potential causative gene for PSL. In pigs, the *HOMER1* might play an important role in glycogen metabolism and muscle development during piglet birth, when there was a transition of slow-oxidative to fast-glycolytic fiber types, and the liver and skeletal muscles served as the first important energy stores [46].

#### JMY

The other candidate gene on SSC2, *JMY,* is located upstream of the LD block and 15.7 kb away from the significant SNP ALGA0014296 (at 89.8 Mb). *JMY* is an important *P53* cofactor and controls actin dynamics in motile cells [47]. In pigs, *JMY* has important roles in oocyte maturation [48] and early embryo development [49]. In consideration of *JMY* as a regulator of actin filament assembly, it might play an important role in controlling and maintaining the shape and internal structure of muscle fibers. Thus *JMY* could be regarded as a candidate gene for PSL and further studies should focus on the investigation of its function in the late fetal or early postnatal period. In addition, *JMY* was reported to be related to severity of ankylosing spondylitis (AS) in Chinese Han patients [50, 51]. AS caused pain and swelling at large limb joints, especially at the knees in human beings. While in prepubescent cases, pain and swelling might also manifest in the ankles and feet. The mortality rate of male patients was significantly (*P* < 0.001) higher than that of female patients [52]. PSL was characterized by an impaired ability to stand and walk after birth [2], and the higher incidence in male piglets was confirmed in both our study and previous investigations [5–7]. Intuitively, it should be reasonable to assume that PSL might be an animal AS disease model affected by *JMY*, such as piglets affected with AS could not stand and walk because of the pain from large limb joints.

#### ITGA1

On SSC16, the nearest gene *ITGA1* is located 94.3 kb upstream of the significant SNPs MARC0006026 (at 33.9 Mb). The *ITGA1* gene encodes the alpha 1 subunit of integrin receptors and could negatively regulate cell proliferation [23]. Its homologous gene *ITGA5* was found differentially expressed in the hind leg muscles between normal and PSL piglets [13]. Moreover, *ITGA* was highly expressed at 35 days-post-coitus in Landrace and 49 days-post-coitus in Lantang (Chinese indigenous obese pig breed), indicating that *ITGA* was important for later muscle differentiation and proliferation [53]. Consequently, the *ITGA1* should be highlighted as a candidate gene related to PSL.

#### RAB32

On SSC1, the nearest gene *RAB32* is located 40 kb upstream of the significant SNP ASGA0001519 (at 21.4 Mb). The *RAB32* encodes an *A-kinase* anchoring protein and participates in both mitochondrial anchoring and dynamics [54]. In addition, *RAB32* was also highlighted as an important potential gene regulating lipid metabolism [55]. Ultrastructural analysis of the muscle of PSL and normal piglets (longissimus dorsi and biceps femoris) clearly demonstrated that mitochondria were often located near the sarcolemma of the normal piglets, but presented within the sarcoplasm of the PSL, which resulted in the increased accumulation of glycogen in the muscles of PSL [11]. It should be noted that glycogen is the first energy store in the piglet at birth. If it could not be properly metabolized, then protein has to be consumed as the substitute, thus finally inducing insufficient energy and higher mortality rate [56]. Furthermore, the total amount of muscle glycogen was several times as great as the total amount of liver glycogen, and most of glycogen was depleted during the first day of life [57]. Mitochondria are particularly important for glycogen and lipid metabolism during the first few days after birth, supporting *RAB32* as a candidate gene for PSL.

#### TNP1 and ENSSSCP00000021921

On SSC15, the two chromosome-wide significant SNPs ALGA0087037 (at 131.9 Mb) and MARC0003725 (at 131.9 Mb) are located in a 22 kb region of moderate LD (r^2^ = 0.45) where no genes are identified. The nearest gene *TNP1* is located 97.5 kb away from the significant SNP ALGA0087037. The *TNP1* gene product plays a major role during spermatogenesis and spermatid differentiation in pig [58], and had an impact on reproductive potential of sperm in mouse [59]. On SSC7,a novel gene *ENSSSCP00000021921* is the nearest to the significant SNP H3GA0022494, however the function of the gene is still not reported. Comparative genomic analysis for 17 eutherian mammals in Ensembl (http://www.ensembl.org) shows that the gene of *ENSSSCP00000021921* is *RAD51B*, which encodes a member of the *RAD51* protein family essential for DNA repair. According to the known function of the genes, it seems unlikely that these genes (on SSC15 and SSC7) are promising loci for SPL.

## Conclusion

In this GWA study, PCA was used to adjust for population structure in order to reduce spurious associations. After strict Bonferroni correction, seven chromosome-wide significant SNPs associated with PSL were identified for the limited sample size and low density SNPs. Those SNPs were located on SSC1, SSC2, SSC7, SSC15, SSC16 and four genes (*HOMER1* and *JMY* on SSC2, *ITGA1* on SSC16, and *RAB32* on SSC1) related to muscle development, metabolism and mitochondrial dynamics were suggested to be the most likely candidate genes for PSL. Further analyses of these loci based on additional genetic and functional studies are expected to reveal the genetic mechanisms responsible for PSL.

## Abbreviations

AS: Ankylosing spondylitis
GWAS: Genome-wide association study
MAF: Minor allele frequency
PC: Principal components
PCA: Principal component analysis
PRRSV: Porcine reproductive and respiratory syndrome virus
PSL: Piglet splay leg syndrome
SSC: *Sus scrofa* chromosome
